# HLA-DQ-Specific Recombinant Human Monoclonal Antibodies Allow for In-Depth Analysis of HLA-DQ Epitopes

**DOI:** 10.3389/fimmu.2021.761893

**Published:** 2022-01-07

**Authors:** Suzanne Bezstarosti, Cynthia S. M. Kramer, Marry E. I. Franke-van Dijk, Manon Vergunst, Kim H. Bakker, Merve Uyar-Mercankaya, Rico Buchli, Dave L. Roelen, Johan W. de Fijter, Frans H. J. Claas, Sebastiaan Heidt

**Affiliations:** ^1^ Department of Immunology, Leiden University Medical Center, Leiden, Netherlands; ^2^ Department of Internal Medicine (Nephrology), Leiden University Medical Center, Leiden, Netherlands; ^3^ Pure Protein LLC, Oklahoma City, OK, United States; ^4^ Eurotransplant Reference Laboratory, Leiden, Netherlands

**Keywords:** monoclonal antibody, human leukocyte antigen (HLA), epitope, eplet, transplantation, amino acid, antibody verification, reactivity pattern

## Abstract

HLA-DQ donor-specific antibodies (DSA) are the most prevalent type of DSA after renal transplantation and have been associated with eplet mismatches between donor and recipient HLA. Eplets are theoretically defined configurations of surface exposed amino acids on HLA molecules that require verification to confirm that they can be recognized by alloantibodies and are therefore clinically relevant. In this study, we isolated HLA-DQ specific memory B cells from immunized individuals by using biotinylated HLA-DQ monomers to generate 15 recombinant human HLA-DQ specific monoclonal antibodies (mAb) with six distinct specificities. Single antigen bead reactivity patterns were analyzed with HLA-EMMA to identify amino acids that were uniquely shared by the reactive HLA alleles to define functional epitopes which were mapped to known eplets. The HLA-DQB1*03:01-specific mAb LB_DQB0301_A and the HLA-DQB1*03-specific mAb LB_DQB0303_C supported the antibody-verification of eplets 45EV and 55PP respectively, while mAbs LB_DQB0402_A and LB_DQB0602_B verified eplet 55R on HLA-DQB1*04/05/06. For three mAbs, multiple uniquely shared amino acid configurations were identified, warranting further studies to define the inducing functional epitope and corresponding eplet. Our unique set of HLA-DQ specific mAbs will be further expanded and will facilitate the in-depth analysis of HLA-DQ epitopes, which is relevant for further studies of HLA-DQ alloantibody pathogenicity in transplantation.

## Introduction

Chronic rejection remains a leading cause of graft loss in long-term renal transplant recipients and is associated with development of *de novo* donor-specific antibodies (dnDSA) (1–3). In order to minimize the chance of developing dnDSA, kidney allocation systems aim to match for human leukocyte antigen (HLA). Most HLA matching algorithms are restricted to HLA-A, -B and -DR on the serological antigen level, whereas dnDSA directed against HLA-DQ are the most prevalent after transplantation and have been associated with rejection, transplant glomerulopathy and allograft loss (4–9).

The induction of dnDSA is caused by mismatched amino acids (AA) on polymorphic sites of the donor HLA molecules. Configurations of surface exposed polymorphic AAs within a 3.0-3.5 Ångstrom (Å) radius have been defined as HLA eplets (10, 11). Multiple studies have demonstrated an association between eplet mismatches and formation of dnDSA (12–17). However, not all theoretically defined eplets are immunogenic and thus verification of actual interactions with antibodies is needed to determine the clinically relevant eplets (18–20). For HLA class I, a large number of eplets has been verified by several different methods (21–27). So far, only a limited number of HLA-DQ eplets have been antibody-verified, mainly due to the lack of proper reagents. Nevertheless, the few currently verified HLA-DQ eplets have been shown to be independently associated with an increased rate of dnDSA occurrence, graft rejection and graft failure (17, 28). However, a residual effect of non-antibody-verified HLA-DQ mismatches on graft loss has also been demonstrated (28), indicating that there are still immunogenic HLA-DQ eplets that have not yet been antibody-verified. Accordingly, identification of clinically relevant HLA-DQ eplets would be instrumental for a better assessment of immunological risk in transplant patients and facilitate personalized medicine such as personalized immunosuppressive drug dosing (16, 29, 30).

Several approaches of antibody verification have been implemented through the years, ranging from serum analysis of uni- and multiparous women, absorption-elution studies, as well as analysis of reactivity patterns of murine and human monoclonal antibodies (mAbs) (20–27, 31–34). Amongst these, we consider human HLA-specific mAbs the most conclusive tool for antibody-verification of theoretically defined eplets (23, 24, 31). Our group recently demonstrated a new method for the generation of HLA-DR specific recombinant human mAbs through isolation of HLA-DR specific memory B cells from peripheral blood utilizing HLA-DR tetramers. Thorough analysis of these mAbs resulted in antibody verification and redefinition of various HLA-DR eplets (35). In this study, we expanded our established techniques to generate novel recombinant human HLA-DQ mAbs by using biotinylated soluble HLA-DQ monomers. Overall, 15 recombinant human HLA-DQ mAbs were produced showing six distinct reactivity patterns in luminex single antigen bead (SAB) analysis. Further analysis of AA sequences of the reactive and non-reactive alleles identified uniquely shared AAs which were mapped to previously defined eplets. To determine the functional epitopes of the mAbs, uniquely shared AAs were identified through analysis of AA sequences of the reactive and non-reactive alleles, which were mapped to previously defined eplets.

## Materials and Methods

### Subjects

Peripheral blood and serum samples from healthy women (N = 5) who developed HLA-DQ antibodies upon pregnancy, were collected with informed consent under guidelines issued by the medical ethics committee of Leiden University Medical Center (Leiden, the Netherlands). Peripheral blood mononuclear cells (PBMC) were isolated by Ficoll-Paque (Pharmacy Leiden University Medical Centre, Leiden, the Netherlands) density gradient centrifugation and kept frozen in liquid nitrogen until further use.

### HLA Typing

Both antibody-producers and HLA immunizers were typed by next-generation sequencing on an Illuminia platform (Illumina, San Diego, CA, USA) using NGSgo kits (GenDx, Utrecht, the Netherlands) as previously described (35). IPD-IMGT/HLA database version 3.35.0 and 3.39.0 were used for analysis.

### Isolation and Expansion of HLA-DQ-Specific Memory B Cells

HLA-DQ-specific memory B cells were isolated and expanded as previously described by Kramer et al. (35) with a few adaptations. From thawed PBMC, B cells were enriched (purity of > 95%) by negative selection using EasySep Human B cell enrichment kits (Stem Cell Technologies, Grenoble, France). The enriched B cells were first incubated with 1 µg of biotinylated soluble HLA-DQ monomers per 2x10^6^ cells (Pure Protein LLC, Oklahoma City, OK, USA) for 30 min at 4°C. Cells were washed with phosphate-buffered saline (PBS) containing 0.1% bovine serum albumin (Sigma-Aldrich, Zwijndrecht, the Netherlands), and stained with streptavidin-PE, streptavidin-APC, mouse anti-human CD3 (Pacific blue, SP34-2), IgD (PE-Cy7, IA6-2) (all from BD Biosciences, Breda, the Netherlands), and CD27 (FITC, CLB-27/1, ThermoFisher Scientific, Waltham, MA, USA) for 30 min at 4°C in the dark. After washing, CD3^-^CD27^+^IgD^-^APC^+^PE^+^ cells were sorted by FACSAria III sorter (BD Biosciences) at 1 cell per well in 96-well flat-bottom plates (Costar, Corning, NY, USA) containing 0.1x10^6^ irradiated CD40L-expressing EL4-B5 cells (36). The memory B cell clones were expanded for 13 days with Iscove’s modified Dulbecco’s medium (IMDM; Gibco Invitrogen, Paisley, UK) supplemented with 10% fetal bovine serum (FBS; Sigma-Aldrich), 2 mM L-glutamine (Gibco), 50 µM 2-mercaptoethanol (Sigma-Aldrich), and 100 U/ml penicillin with 100 µg/ml streptomycin (Gibco), 0.5 µg/ml R848 (toll-like receptor 7/8 agonist, resiquimod), 20 µg/ml insulin-transferrin-sodium selenite (both from Sigma-Aldrich), 50 ng/ml IL-21 (Gibco), 1 ng/ml IL-1β, and 0.3 ng/ml TNFα (both from Miltenyi, Leiden, the Netherlands) (37).

### HLA-Specific Antibody Detection

The supernatant of the memory B cell clones or recombinant human HLA-specific mAbs were screened for the presence of IgG by enzyme-linked immunosorbent assay, as previously described (38). Subsequently, the IgG-positive supernatants were tested with Lifecodes Lifescreen Deluxe screening kit (Immucor Transplant Diagnostics, Stamford, CT, USA) to detect HLA antibodies. The specificities of HLA antibodies were determined by Lifecodes HLA class II SAB assays (Immucor). Serum samples were treated with ethylenediaminetetraacetic acid (6% EDTA) prior to testing. **Supplementary Table 1** lists the alleles present in the SAB panel that was used. HLA antibody data was analyzed with Match It! Antibody software version 1.3.0 (Immucor), with positive HLA alleles assigned according to the software.

### Production of Recombinant Human Monoclonal HLA Antibodies

RNA from HLA-antibody positive memory B cell clones was isolated using TRIzol (ThermoFisher Scientific). Subsequently, human mAbs were generated using recombinant technology as previously described (39). Briefly, genes encoding the variable heavy and light chains were obtained by SMART cDNA synthesis and 5’-RACE polymerase chain reaction, which were then cloned into pcDNA3.3 expression containing the corresponding human constant domain IgG1, kappa or lambda. Next, plasmids of the heavy and light chain were generated and used for transient co-transfection of ExpI293F cells (ThermoFisher Scientific) with SV40-LT plasmid (40), ExpiFectamine, Opti-Mem, Expi293 expression medium (all ThermoFisher Scientific) to produce recombinant human mAbs. Sanger sequencing (Macrogen, Amsterdam, the Netherlands) of heavy and light chain plasmids was performed to obtain nucleotide sequence data of the variable domain. Sequence data was analyzed with IgBLAST (41) to define V(D)J gene usage.

### Reactivity Analysis of Recombinant Human HLA-DQ mAbs

HLA-EMMA version 1.05 was used to determine the solvent accessible AA mismatches between the HLA-DQ alleles of the antibody producer and the mismatched HLA-DQ allele(s) of the immunizer, or the HLA-DQ monomer that was used for memory B cell sorting (42). Next, it was determined whether these solvent accessible AA mismatches were uniquely shared by the reactive HLA alleles and absent on the non-reactive HLA alleles. In order to visualize AA positions and to establish whether AAs were within a 3.5 Å or 15 Å radius to form a functional or structural epitope respectively, the following HLA-DQ crystal structures were visualized in Swissviewer (43): Protein Data Bank (PBD) 1S9V, 4Z7U and 1UVQ (downloaded from https://www.rcsb.org/on February 24, 2021). Antibody reactivity patterns of the mAbs were analyzed with HLAMatchmaker (DRDQDP Antibody Analysis Program v3.1; http://www.epitopes.net/) to identify eplets present on the reactive HLA alleles. Polymorphic residue definition and antibody-verification status of eplets were extracted from the HLA Epitope Registry (http://www.EpRegistry.com.br accessed on May 6, 2021).

### Cells

HLA-typed Epstein-Barr virus-transformed lymphoblastoid cell lines (EBV-LCLs) were cultured in IMDM supplemented with 10% FBS, 2 mM L-glutamine, 50 µM 2-mercaptoethanol, and 100 U/ml penicillin with 100 µg/ml streptomycin.

### Flow Cytometric Crossmatch Assay (FACS-XM)

As previously described (35), EBV-LCLs were first incubated with mAb (20 µg/ml) or PBS at room temperature (RT) for 30 min. After washing, the cells were stained with mouse anti-human CD3 (PE, SK7), CD19 (APC, HIB19, both from BD Bioscience), and rabbit anti-human IgG F(ab’)2 (FITC, Dako, Leiden, the Netherlands) at 4°C in the dark for 30 min. Next, the washed cells were fixed with 1% paraformaldehyde (Pharmacy LUMC) and acquired using Accuri C6 flow cytometry (BD Bioscience). Data were analyzed using FlowJo V10 software (Ashland, OR, USA).

### Complement-Dependent Cytotoxicity Assay

Complement-dependent cytotoxicity (CDC) assays were performed as previously described (35). EBV-LCLs were incubated with various concentrations of mAbs (0.3125 - 0.625 - 1.25 - 2.5 - 5 - 10 - 20 - 40 µg/ml) or PBS in Terasaki plates (Greiner, Frickenhausen, Germany) for 60 min at RT. Cells were then incubated for another 60 min at RT with 5 µl rabbit complement (Inno-train, Kronberg, Germany). Cytotoxicity of EBV-LCLs was visualized after 15 min in the dark at RT incubation with 5 µl propidium iodide ink (Sigma-Aldrich), and analyzed with the Patimed (Leica Microsystems, Amsterdam, the Netherlands).

## Results

### Recombinant Human HLA-DQ-Specific mAbs Generated From HLA-Specific Memory B Cell Clones

A total of 15 recombinant human HLA-DQ-specific mAbs (**Table 1**) were generated from unique HLA-DQ-specific memory B cell clones isolated from five individuals using six different HLA-DQ monomers (**Table 2**). HLA specificity in the serum of the immunized subjects and HLA specificity of the generated HLA-DQ-specific mAbs was confirmed by SAB analysis (**Supplementary Figure 1**), which demonstrated that all mAbs were directed against the beta chain of the HLA-DQ molecule and none were cross-reactive against HLA-DR or HLA-DP alleles. These findings were further supported by FACS-XM and CDC assays which confirmed binding to the natively expressed target-HLA molecule and their cytotoxic capacity (**Supplementary Figures 2, 3**). When multiple mAbs with identical reactivity patterns were generated from one individual, only one mAb will be discussed in detail.

**Table 1:** Information on HLA-DQ monoclonal antibodies and reactive HLA-DQB1 alleles.

					On reactive HLA-DQB1 Alleles		
Human mAb	HLA-DQ Antibody Producer	HLA Immunizer	HLA Monomer	Reactive HLA-DQB1 Alleles	HLAMatchmaker Version 3.1 eplet	HLA Epitope Registry Eplet	Uniquely Shared Residues
**LB_DQB0201_A** ^1^ LB_DQB0201_B LB_DQB0201_C	*Individual #1* DQB1*03:01^2^, DQB1*06:02, DQA1*01:02, DQA1*05:05		DQB1*02:01/DQA1*02:01	DQB1*02:01, DQB1*02:02	52LL (AbVer)	52LL (52L 55L 28S 30S 37I)	46E, 52L, 55L, 71K, 74A
**LB_DQB0301_A** LB_DQB0301_B LB_DQB0301_C	*Individual #2* DQB1*03:02, DQB1*04:02, DQA1*03:01, DQA1*03:03	DQB1*03:01, DQB1*04:02, DQA1*03:03, DQA1*05:05	DQB1*03:01/DQA1*02:01	DQB1*03:01	45EV (AbVer)	45EV (45E 46V 47Y)	45E
**LB_DQB0303_A**	*Individual #3* DQB1*06:02, DQB1*06:03, DQA1*01:02, DQA1*01:03	DQB1*03:01^3^, DQB1*06:03, DQA1*01:03, DQA1*03:03	DQB1*03:03/DQA1*02:01	DQB1*02:01, DQB1*02:02, DQB1*03:01, DQB1*03:02, DQB1*03:03, DQB1*04:01, DQB1*04:02	84QL (AbVer) 125AQ (other)	84QL (84Q 86E 87L 89T 90T 125A)	53L, 84Q, 85L, 86E, 87L, 89T, 90T, 125A, 220H, 221H
**LB_DQB0303_B**	*Individual #3* DQB1*06:02, DQB1*06:03, DQA1*01:02, DQA1*01:03	DQB1*03:01^3^, DQB1*06:03, DQA1*01:03, DQA1*03:03	DQB1*03:03/DQA1*02:01	DQB1*03:01, DQB1*03:02, DQB1*03:03, DQB1*04:01, DQB1*04:02	52PL (AbVer) 182N (other)	182N	52P+53L, 140T, 182N
**LB_DQB0303_C** LB_DQB0303_D	*Individual #3* DQB1*06:02, DQB1*06:03, DQA1*01:02, DQA1*01:03	DQB1*03:01^3^, DQB1*06:03, DQA1*01:03, DQA1*03:03	DQB1*03:03/DQA1*02:01	DQB1*03:01, DQB1*03:02, DQB1*03:03	55PP (AbVer)	55PP (55P 56P)	55P
**LB_DQB0402_A** LB_DQB0402_B LB_DQB0402_C	*Individual #4* DQB1*03:01^2^, DQB1*03:01^3^, DQA1*03:03^4^, DQA1*05:05/ DQA1*05:11	DQB1*03:01^2^, DQB1*06:02, DQA1*01:02, DQA1*05:05	DQB1*04:02/DQA1*02:01	DQB1*04:01, DQB1*04:02, DQB1*05:01, DQB1*05:02, DQB1*05:03, DQB1*06:01, DQB1*06:02, DQB1*06:03, DQB1*06:04	-	55R	55R
**LB_DQB0601_B**	*Individual #4* DQB1*03:01^2^, DQB1*03:01^3^, DQA1*03:03^4^, DQA1*05:05/DQA1*05:11	DQB1*03:01^2^, DQB1*06:02, DQA1*01:02, DQA1*05:05	DQB1*06:01/DQA1*02:01	DQB1*04:01, DQB1*04:02, DQB1*05:01, DQB1*05:02, DQB1*05:03, DQB1*06:01, DQB1*06:02, DQB1*06:03, DQB1*06:04	-	55R	55R
**LB_DQB0602_B**	*Individual #5* DQB1*02:01^5^, DQB1*03:01, DQA1*05:01, DQA1*05:05		DQB1*06:02/DQA1*01:01	DQB1*04:01, DQB1*04:02, DQB1*05:01, DQB1*05:02, DQB1*05:03, DQB1*06:01, DQB1*06:02, DQB1*06:03, DQB1*06:04	–	55R	55R

^1^Reactivity analyses of mAbs in bold are discussed.

^2^Typing is DQB1*03:01/03:297; these alleles do not have amino acid mismatches.

^3^Typing is DQB1*03:01/03:276N/03:292; DQB1*03:292 has 1 solvent-accessible amino acid mismatch on position 214.

^4^Typing is DQA1*03:03/03:11; these alleles have 1 not solvent-accessible amino acid mismatch on position 199.

^5^Typing is DQB1*02:01/02:109; these alleles have 1 solvent-accessible amino acid mismatch on position 204.

mAb, monoclonal antibody; AbVer, antibody-verified.

**Table 2:** V(D)J usage of the different HLA-DQ monoclonal antibodies.

Subject	Clone	Heavy chain					Light chain				
		V gene	J gene	D gene	CDR3	Identity (%)	κ or λ	V gene	J gene	CDR3	Identity (%)
#1	LB_DQB0201_A	4-30-4	3	2-15	AREYCSGASCYRDAFDI	97.3	λ	3-1	1	QAWDSSIGV	97.9
	LB_DQB0201_B	4-4	1	1-26	ARDSIVGATIVGYFQH	96.6	κ	2-29	4	MQGIHLPPT	97.4
	LB_DQB0201_C	3-30	6	4-17	ARDIGWATETTGYFYGLDV	95.9	λ	2-18	1	SSYTSSSTYV	98.6
#2	LB_DQB0301_A	3-7	4 or 5^1^	5-12	GRVYSRGYSGYM	95.9	κ	3-11	4	QQRSDWPPGLT	98.3
	LB_DQB0301_B	4-39	6	2-21	ARQSRSMRGCGGACWGKYGMDV	97.0	κ	3-20	4	QQYGSSPLT	97.6
	LB_DQB0301_C	1-69	4	6-13	AREGGGSSSWYSD	93.9	κ	3-20	1	QQYGNSPRT	96.5
#3	LB_DQB0303_A	3-48	4	5-12	ARDHAHIEATGPLDC	93.9	κ	1-5	3	QQYFSYPGFT	98.2
	LB_DQB0303_B	4-30-2	4	1-26	ARDTGAEGYFDF	96.3	κ	1-12	4	QQADSFPRT	95.5
	LB_DQB0303_C	4-30-4	4 or 5^1^	3-22	ARDGYDSSGAF	94.6	κ	1-39 or 1D-39^1^	2	QQSYSPPHT	95.9
	LB_DQB0303_D	3-23 or 3-23D^1^	4 or 5^1^	3-22	AKYKTGSYYDNTGYNPLPDS	94.6	κ	4-1	1	QQYYTTPWT	98
#4	LB_DQB0402_A	1-18	4	6-6	AREMSSSSSLIDY	96.3	κ	1-5	1	QQYNNYSPTT	95.8
	LB_DQB0402_B	5-51	3	2-21 or 3-10^1^	AMWSGSTNDAFDI	94.8	λ	3-10	3	YSTDSSGHHWV	98.6
	LB_DQB0402_C	3-15	3	3-22	TTDSSLGAYDSSGYYYVGAFDI	97.4	λ	1-39 or 1D-39^1^	2	QQSYSTPYT	96.8
	LB_DQB0601_B	4-61	4	5-24	ARDNFNYYFDL	95.7	λ	1-44	2 or 3^1^	AAWDDSLNGLV	95.6
#5	LB_DQB0602_B	3-33	6	4-11 or 4-4^1^	AREPEPTLTTLHYYYAMDV	97.0	κ	3-20	2	QQFATSPMYT	96.9

^1^Multiple equivalent top matches.

### Reactivity Analysis of mAb LB_DQB0201_A

**Figure 1:**
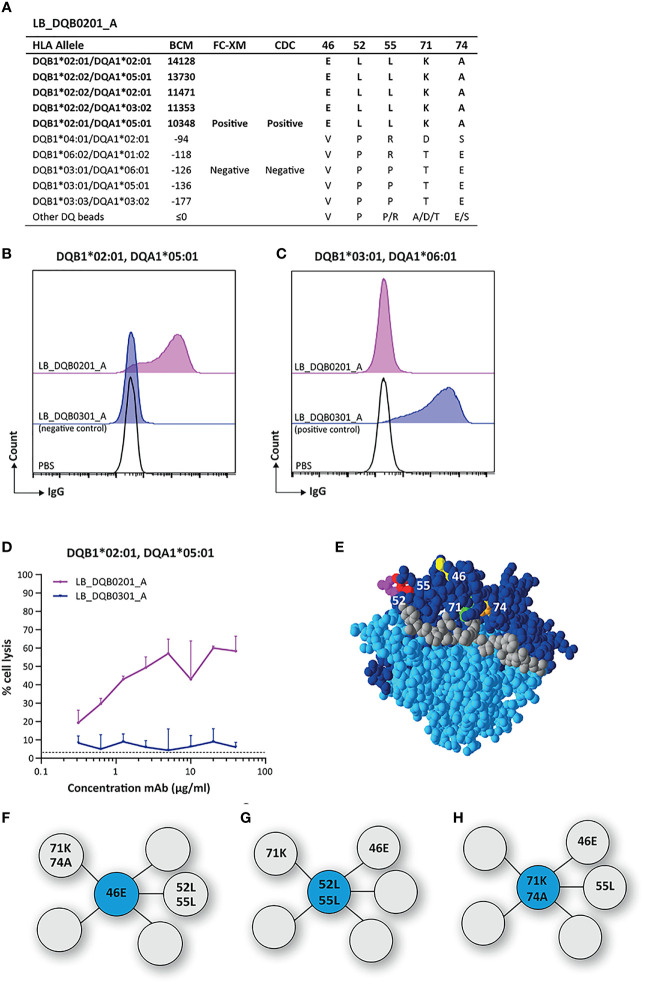
Reactivity analysis of monoclonal antibody LB_DQB0201_A. **(A)** Comparison of the amino acid positions of interest of the HLA-DQB1 alleles on the reactive beads in the single antigen bead assay and a selection of the non-reactive HLA-DQB1 alleles. Flow cytometry crossmatches on EBV-LCLs expressing DQB1*02:01 **(B)** and DQB1*03:01 **(C)** showed that LB_DQB0201_A binds to natively expressed DQB1*02:01, but not to DQB1*03:01. **(D)** LB_DQB0201_A induced complement dependent cell lysis in a dose-dependent manner for cells expressing DQB1*02:01. **(E)** Locations of amino acid 46E (yellow), 52L (magenta), 55L (red), 71K (green) and 74A (orange) are indicated on the crystal structure of DQB1*02:01/DQA1*05:01 (PBD: 1S9V). The β chain is depicted in dark blue, the α chain in light blue, and the peptide in grey. **(F)** Schematic representation of the antibody-footprint of LB_DQB0201_A with 46E or **(G)** 52L+55L or **(H)** 71K+74A as the functional epitope (cyan) with additional AA configurations (grey). Monoclonal antibody concentrations used for testing were 20 µg/ml for single antigen bead assay and FC-XM and 0.3125 - 0.625 - 1.25 - 2.5 - 5 - 10 - 20 - 40 µg/ml for CDC. BCM, background corrected mean fluorescence intensity; FC-XM, flowcytometric crossmatch; CDC, complement dependent cytotoxicity; PBS, phosphate-buffered saline; mAb, monoclonal antibody; EBV-LCL, Epstein-Barr Virus-transformed lymphoblastoid B-cell line; PBD, Protein Data Bank.

The mAb LB_DQB0201_A displayed a narrow reactivity pattern in the SAB assay, solely recognizing DQB1*02:01 and DQB1*02:02 (**Figure 1A**). We confirmed the binding of LB_DQB0201_A to its physiologically expressed HLA target in a FACS-XM and its cytotoxicity capacity in a CDC assay with EBV-LCLs expressing DQB1*02:01 (**Figures 1B–D**). Next, HLA-EMMA was used to determine AA mismatches between DQB1*03:01 and DQB1*06:02 of the antibody-producer and DQB1*02:01 of the HLA-DQ monomer used for cell sorting, as the immunizer’s HLA type was unknown. Comparison of the AA sequences of DQB1*02:01 and DQB1*02:02 with the non-reactive HLA-DQB1 alleles identified five of the AA mismatches to be uniquely shared by the reactive HLA-DQB1 alleles in the SAB assay. These residues, glutamic acid (E) on position 46, leucine (L) on position 52 and position 55, lysine (K) on position 71 and alanine (A) on position 74 (**Figure 1A**) are all located on the top of the HLA-DQB1*02:01/DQA1*05:01 molecule (**Figure 1E**). HLAMatchmaker analysis demonstrated that the reactive HLA-DQB1 alleles shared eplet 52LL (52L 55L 28S 30S 37I), which comprises two of the five uniquely shared AAs that were identified with HLA-EMMA. Although uniquely shared by the reactive HLA-DQB1 alleles, we did not consider the other three residues (28S 30S 37I), because these positions have not been defined as solvent accessible by HLA-EMMA and thus cannot be accessed by the B cell receptor to induce an antibody response. While HLAMatchmaker identified only eplet 52LL, we observed three possible functional epitopes for LB_DQB0201_A; 46E (**Figure 1F**), or 52L + 55L (**Figure 1G**), or 71K + 74A (**Figure 1H**). For each functional epitope, at least two or more of the other unique residues are located within a 15 Å radius. As the five uniquely shared residues are always present together on the HLA-DQB1 alleles represented in the currently available SAB assays, it is not possible to determine which of the functional epitopes induced the antibody response.

### Reactivity Analysis of mAb LB_DQB0301_A

**Figure 2:**
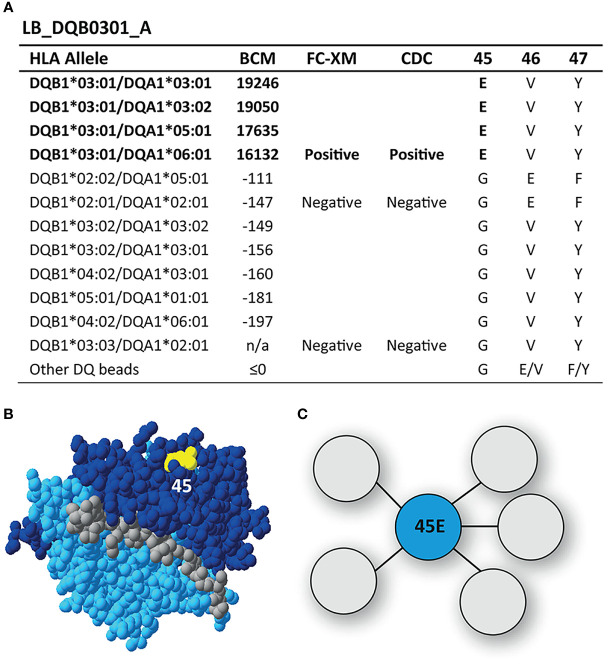
Reactivity analysis of monoclonal antibody LB_DQB0301_A. **(A)** Comparison of the amino acid positions of interest of the HLA-DQB1 alleles on the reactive beads in the single antigen bead assay and a selection of the nonreactive HLA-DQB1 alleles. **(B)** Location of amino acid 45E (yellow) is indicated on the crystal structure of DQB1*03:02/DQA1*03:01 (PBD: 4Z7U). The β chain is depicted in dark blue, the α chain in light blue, and the peptide in grey. **(C)** Schematic representation of the antibody-footprint of LB_DQB0301_A interacting with the functional epitope 45E (cyan). BCM, background corrected mean fluorescence intensity; FC-XM, flowcytometric crossmatch; CDC, complement dependent cytotoxicity; PBD, Protein Data Bank.

SAB analysis of mAb LB_DQB0301_A showed only reactivity against the immunizing allele DQB1*03:01, which was also confirmed with cellular assays (**Figure 2A**). Upon comparing the AA sequences of reactive and non-reactive HLA-DQB1 alleles in the SAB assay, 45E was identified to be unique for DQB1*03:01. This residue is located on the top of the molecule (**Figure 2B**) and is the functional epitope of LB_DQB0301_A (**Figure 2C**). While the antibody-verified eplet 45EV (45E 46V 47Y) was identified by HLAMatchmaker analysis as uniquely present on DQB1*03:01, residues 46V and 47Y are self amino acids for the antibody-producer and are both present on non-reactive HLA-DQB1 alleles. Further, residue 47Y is defined as not solvent accessible by HLA-EMMA. Based on the SAB analysis of mAb LB_DQB0301_A however, it is not possible to determine whether residues 46V and 47Y are crucial for antibody-induction or that 45E is sufficient and should be considered as the eplet. Since we cannot rule out that 46V and 47Y are part of the functional epitope and the corresponding eplet, we conclude that mAb LB_DQB0301_A supports the antibody-verification of eplet 45EV.

### Reactivity Analysis of mAb LB_DQB0303_A

**Figure 3:**
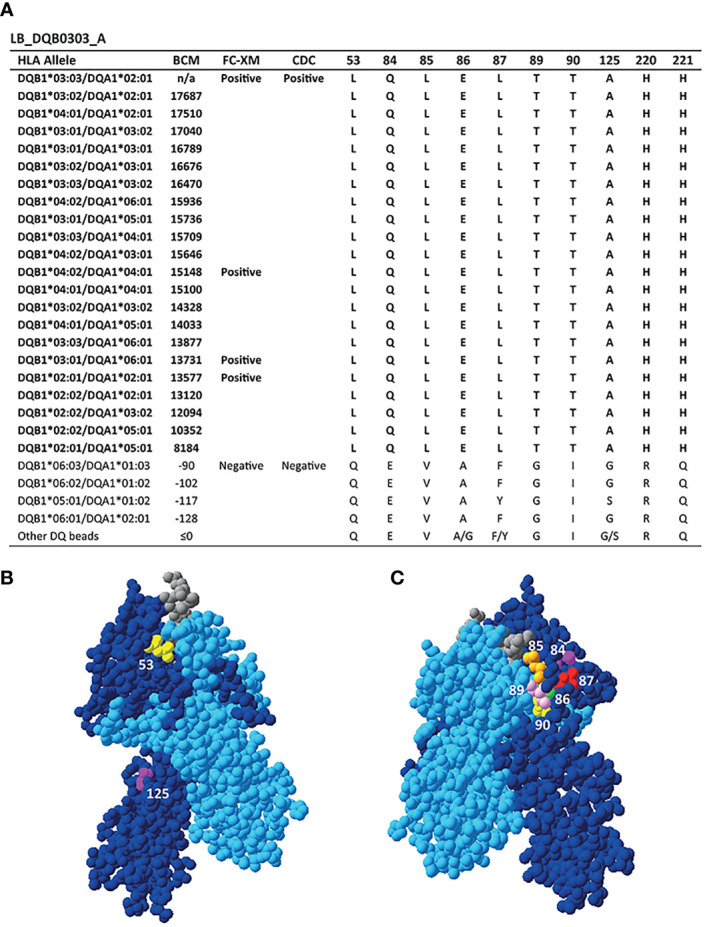
Reactivity analysis of monoclonal antibody LB_DQB0303_A. **(A)** Comparison of the amino acid positions of interest of the HLA-DQB1 alleles on the reactive beads in the single antigen bead assay and a selection of the non-reactive HLA-DQB1 alleles. HLA-DQB1*03:03/DQA1*02:01 was not present in the single antigen bead panel. Locations of amino acid 53L (yellow), 125A (magenta) **(B)** and 84Q (magenta), 85L (orange), 86E (green), 87L (red), 89T (pink) and 90T (yellow) **(C)** are indicated on the crystal structure of DQB1*03:02/DQA1*03:01 (PBD: 4Z7U). The position of residues 220H and 221H could not be determined, as these AAs are not included in the crystal structure. The β chain is depicted in dark blue, the α chain in light blue, and the peptide in grey. BCM, background corrected mean fluorescence intensity; FC-XM, flowcytometric crossmatch; CDC, complement dependent cytotoxicity; PBD, Protein Data Bank.

A more broadly reactive HLA-DQ-specific mAb is LB_DQB0303_A, which binds to DQB1*02:01, DQB1*02:02, DQB1*03:01, DQB1*03:02, DQB1*03:03, DQB1*04:01 and DQB1*04:02 in the SAB assay (**Figure 3A**). These alleles uniquely share 10 residues, namely L on position 53, glutamine (Q) on position 84, L on position 85, E on position 86, L on position 87, threonine (T) on position 89, T on position 90, A on position 125, histidine (H) on position 220 and H on position 221. Six of these AAs are present within eplet 84QL (84Q 86E 87L 89T 90T 125A), which also was one of the eplets identified by HLAMatchmaker to be solely present on the reactive HLA-DQB1 alleles. However, the residues constituting eplet 84QL exceed the 3.5 Å radius and therefore cannot form a single eplet. Interestingly, eplet 125AQ was also identified, but this configuration is not further specified in HLAMatchmaker and is not present in the Epitope Registry either. Because of the relatively high number of uniquely shared residues and the positions of the AAs involved (**Figures 3B, C**), there are many different possible configurations of the functional epitope of LB_DQB0303_A. Without additional data on the actual interaction between LB_DQB0303_ A antibody and the HLA molecule it is not possible to define the functional epitope that triggered the antibody response.

### Reactivity Analysis of mAb LB_DQB0303_B

**Figure 4:**
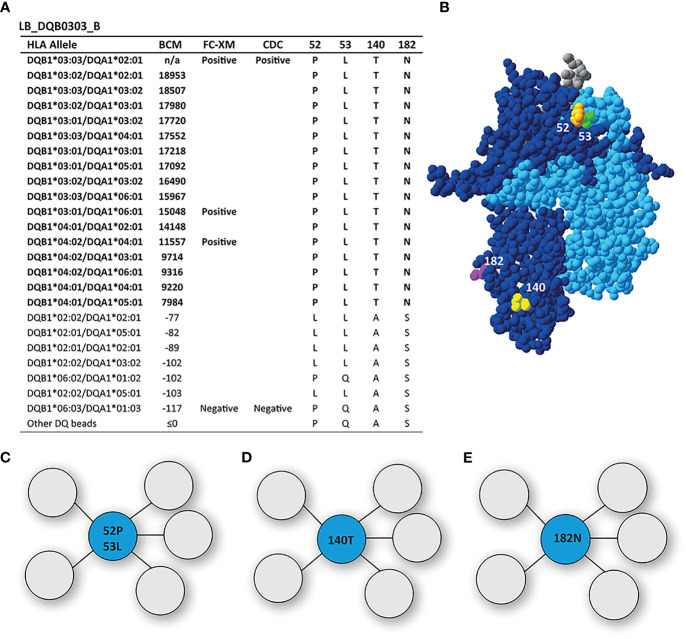
Reactivity analysis of monoclonal antibody LB_DQB0303_B. **(A)** Comparison of the amino acid positions of interest of the HLA-DQB1 alleles on the reactive beads in the single antigen bead assay and a selection of the nonreactive HLA-DQB1 alleles. HLA-DQB1*03:03/DQA1*02:01 was not present in the single antigen bead panel. **(B)** Locations of amino acid 52L (orange), 53L (green), 140T (yellow) and 182N are indicated on the crystal structure of DQB1*03:02/DQA1*03:01 (PBD: 4Z7U). The β chain is depicted in dark blue, the α chain in light blue, and the peptide in grey. **(C)** Schematic representation of the antibody footprint of LB_DQB0303_B interacting with 52P+53L, or 140T **(D)** or 182N **(E)** as the functional epitope (cyan). BCM, background corrected mean fluorescence intensity; FC-XM, flowcytometric crossmatch; CDC, complement dependent cytotoxicity; PBD, Protein Data Bank.

The mAb LB_DQB0303_B was generated from the same individual as LB_DQB0303_A, but showed a more restricted reactivity pattern against DQB1*03:01, DQB1*03:02, DQB1*03:03, DQB1*04:01 and DQB1*04:02 (**Figure 4A**). The uniquely shared AA residues of these HLA-DQB1 alleles are T on position 140 and asparagine (N) on position 182, of which the latter was also identified by HLAMatchmaker. Interestingly, HLAMatchmaker also identified 52PL (52P 53L); an eplet that is not present anymore in the current version of the HLA Epitope Registry. Although neither 52P nor 53L are uniquely shared between DQ3 and DQ4, and 52P is present on the antibody producer’s HLA, the combination of 52P and 53L is indeed uniquely shared by the reactive HLA-DQB1 alleles. While residues 52P and 53L are located within a 3.5 Å radius on top of the DQB1*03:02/DQA1*03:01 molecule, residues 140T and 182N are located near the transmembrane region and are too far apart to form either a functional or structural epitope together (**Figures 4B–E**). Hence, mAb LB_DQB0303_B is specific for one of three possible functional epitopes; 52P+53L, or 140T, or 182N.

### Reactivity Analysis of mAb LB_DQB0303_C

**Figure 5:**
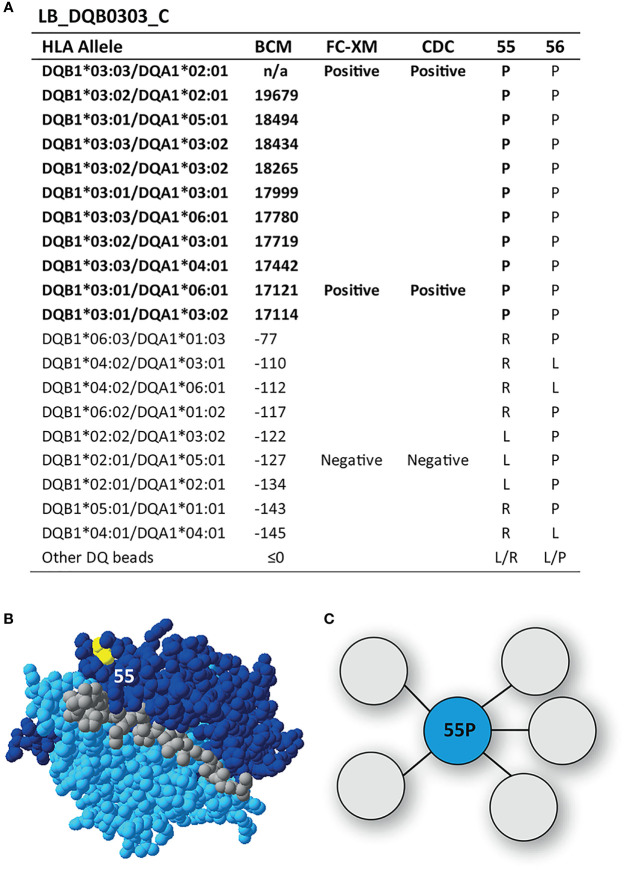
Reactivity analysis of monoclonal antibody LB_DQB0303_C. **(A)** Comparison of the amino acid positions of interest of the HLA-DQB1 alleles on the reactive beads in the single antigen bead assay and a selection of the nonreactive HLA-DQB1 alleles. HLA-DQB1*03:03/DQA1*02:01 was not present in the single antigen bead panel. **(B)** Location of amino acid 55P (yellow) is indicated on the crystal structure of DQB1*03:02/DQA1*03:01 (PBD: 4Z7U). The β chain is depicted in dark blue, the α chain in light blue, and the peptide in grey. **(C)** Schematic representation of the antibody footprint of LB_DQB0303_C interacting with 55P as the functional epitope (cyan). BCM, background corrected mean fluorescence intensity; FC-XM, flowcytometric crossmatch; CDC, complement dependent cytotoxicity; PBD, Protein Data Bank.

The mAb LB_DQB0303_C was reactive against DQB1*03:01, DQB1*03:02 and DQB1*03:03, demonstrating a more narrow reactivity pattern than mAbs LB_DQB0303_A and LB_DQB0303_B, which were generated from the same individual (**Figure 5A**). The DQB1*03 alleles share residue proline (P) on position 55, which is located on top of the DQB1*03:02/DQA1*03:01 molecule (**Figure 5B**) and is the functional epitope of LB_DQB0303_C (**Figure 5C**). Residue 55P is part of eplet 55PP (55P 56P) which was identified by HLAMatchmaker and also includes residue 56P. This residue is not a mismatched amino acid for the antibody producer and is also present on non-reactive HLA-DQB1 alleles. However, it is not possible to determine whether or not 56P is crucial for antibody induction. Therefore, we cannot rule out that 56P is part of the eplet and we conclude that mAb LB_DQB0303_C supports the antibody verification of eplet 55PP.

### Reactivity Analysis of mAbs LB_DQB0402_A, LB_DQB0601_B and LB_DQB0602_B

**Figure 6:**
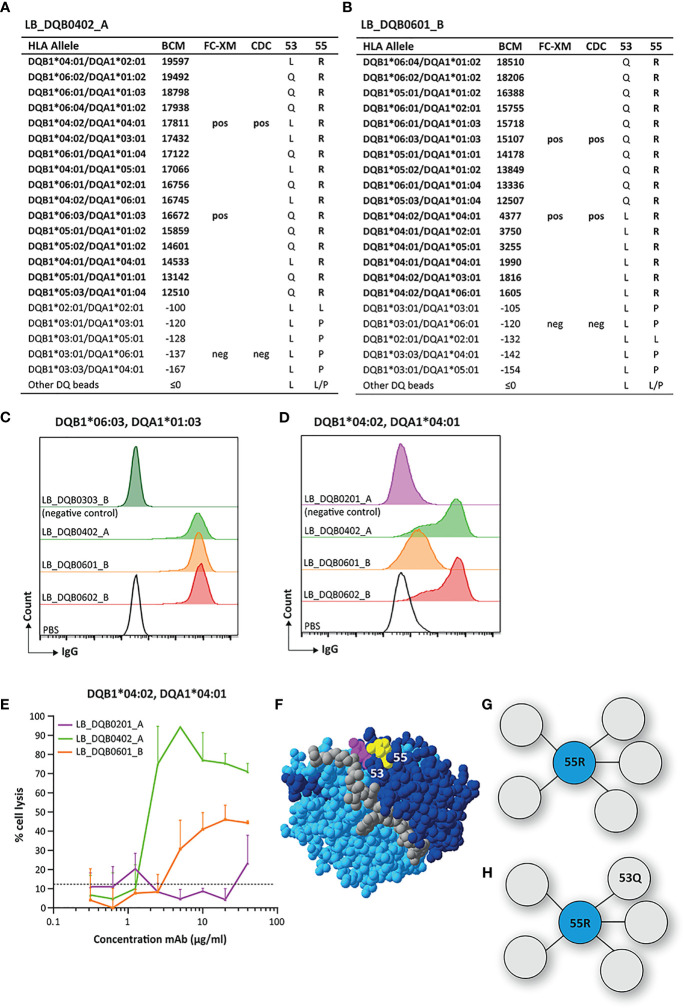
Reactivity analysis of monoclonal antibodies LB_DQB0402_A and LB_DQB0601_B. **(A)** Comparison of the amino acid positions of interest of the HLA-DQB1 alleles on the reactive beads in the single antigen bead assay and a selection of the nonreactive HLA-DQB1 alleles for LB_DQB0402_A and LB_DQB0601_B **(B)**. Flow cytometry crossmatches with EBV-LCLs showed a higher binding strength of mAb LB_DQB0601_B for DQB1*06:03-expressing cells **(C)** than DQB1*04:02-expressing cells **(D)**. **(E)** LB_DQB0402_A induced a higher percentage of complement dependent cell lysis than LB_DQB0601_B for DQB1*04:02-expressing cells. **(F)** Locations of amino acid 53Q (magenta) and 55R (yellow) are indicated on the crystal structure of DQB1*06:02/DQA1*01:02 (PBD: 1UVQ). The β chain is depicted in dark blue, the α chain in light blue, and the peptide in grey. **(G)** Schematic representation of the footprint of mAbs LB_DQB0402_A and LB_DQB0602_B interacting with functional epitope 55R (cyan). **(H)** Schematic representation of the antibody footprint of LB_DQB0601_B that has a stronger binding to the functional epitope 55R (cyan) when additional amino acid 53Q (grey) is present. Monoclonal antibody concentrations used for testing were 20 µg/ml for single antigen bead assay and FC-XM and 0.3125 - 0.625 - 1.25 - 2.5 - 5 - 10 - 20 - 40 µg/ml for CDC. BCM, background corrected mean fluorescence intensity; FC-XM, flowcytometric crossmatch; CDC, complement dependent cytotoxicity; pos, positive; neg, negative; PBS, phosphate-buffered saline; mAb, monoclonal antibody; EBV-LCL, Epstein-Barr Virus-transformed lymphoblastoid B-cell line; PBD, Protein Data Bank.

The mAbs LB_DQB0402 and LB_DQB0601_B were generated from the same individual using HLA-DQB1*04:02 and HLA-DQB1*06:01 monomers, respectively, and showed similar reactivity patterns against HLA alleles DQB1*04:01, DQB1*04:02, DQB1*05:01, DQB1*05:02, DQB1*05:03, DQB1*06:01, DQB1*06:02, DQB1*06:03 and DQB1*06:04 in SAB analysis (**Figures 6A, B**). LB_DQB0602_B was generated from a different individual with a HLA-DQB1*06:02 monomer but showed the same reactivity pattern (**Supplementary Figure 4**). The HLA-DQB1 alleles recognized by these three mAbs all shared arginine (R) on position 55. Interestingly, LB_DQB0601_B showed stronger binding for HLA antigens with 53Q, which is not only illustrated by higher mean fluorescence intensity (MFI) in SAB analysis, but also by the FACS-XM and CDC results. FACS-XM of LB_DQB0601_B with EBV-LCLs carrying DQB1*06:03, an allele that bears 53Q, showed a significantly higher MFI than with DQB1*04:02-carrying cells that lack this residue (**Figures 6C, D**). Accordingly, cell lysis by LB_DQB0601_B was also substantially lower in CDC assays with cells expressing DQB1*04:02 than DQB1*06:03-expressing cells (**Figures 6E** and **Supplementary Figure 3D**). This difference in binding strength and cytotoxicity was not observed for mAbs LB_DQB0402_A and LB_DQB0602_B (**Figures 6C–E** and **Supplementary Figure 3D**) and could not be explained by the alpha chain of the HLA-DQ molecules. As 55R is uniquely shared by all reactive HLA-DQB1 alleles, we consider this residue as the functional epitope for mAbs LB_DQB0402_A and LB_DQB0602_B (**Figures 6F, G**), which further supports the already antibody-verified eplet 55R in the HLA Epitope Registry. Surprisingly, this nor any other eplet was identified by HLAMatchmaker. Although residue 53Q, which is located within a 3.5 Å distance of 55R, is not crucial for antibody binding, it evidently affects the binding strength of mAb LB_DQB0601_B (**Figures 6F, H**). Whether residue 53Q is part of the functional epitope for mAb LB_DQB0601_B is yet to be determined.

## Discussion

In this study, we generated 15 recombinant human HLA-DQ specific mAbs with six distinct specificities through isolation of memory B cells from immunized individuals using biotinylated soluble HLA-DQ monomers. Whereas previously HLA tetramers were used for memory B cell isolation, this study shows that also monomeric HLA molecules can be used for HLA-specific memory B cell isolation. Although antibodies specific for the HLA-DQ alpha chain (DQA1) have been described (32, 44, 45), analysis of SAB assay reactivity patterns demonstrated that all mAbs were only specific for the HLA-DQ beta chain (DQB1). This is likely to be explained by the HLA-DQ monomers used for isolation, which contained HLA-DQA1 alleles that did not correspond to any of the immunizing alleles of our subjects. Since only mismatched eplets on the alpha or beta chain of the donor HLA induce the formation of antibodies directed towards HLA alleles bearing that particular eplet (10), it is not surprising that only HLA-DQB1-specific memory B cells were sorted. While our generated mAbs are directed against HLA-DQB1 only, it has been previously described that structural epitopes on HLA-DQ molecules can cover both the alpha and beta chain (44, 46). Additionally, DSA specific for a particular combination of HLA-DQA1 and HLA-DQB1 have been reported, suggesting that the functional epitope and/or any additional crucial configurations can consist of residues on both chains (47). Reactivity pattern analysis of the mAbs was limited due to the restricted number of HLA-DQA1/HLA-DQB1 combinations in the SAB assay. Additional testing of the mAbs in a SAB assay from another vendor (One Lambda) would expand the number of HLA-DQA1/HLA-DQB1 combinations tested. However, since it is unlikely that the mAbs presented here are specific for the HLA-DQA1/HLA-DQB1 combination, as all beads containing a particular HLA-DQB1 allele were positive regardless of the HLA-DQA1 allele, these analyses were not performed. Nonetheless, the proximity of HLA-DQA1 to several of the uniquely shared AAs on HLA-DQB1 does suggest that both chains could play a role in the structural epitopes recognized by the mAbs. Selection of patient samples that display serum reactivity specifically against HLA-DQA1 and the increasing availability of soluble HLA-DQ monomers will enhance the likelihood of generating mAbs directed against previously described HLA-DQA1 eplets (32, 44, 45) or mAbs specific for a HLA-DQA1/HLA-DQB1 combination (47).

The original definition of an eplet resembles the functional epitope, which determines the antibody specificity through its interaction with the complementarity-determining region 3 of the heavy chain (CDR-H3) of the antibody (11, 48–50). In order to determine the functional epitopes of our generated mAbs, we used HLA-EMMA to identify uniquely shared AAs of the reactive HLA alleles which we then correlated to known eplets. Similar to the previously described analyses of HLA-DR specific mAbs (35), the analysis of mAbs LB_DQB0301_A and LB_DQB0303_C supported the antibody-verified status of eplet 45EV and eplet 55PP respectively.

For mAbs LB_DQB0201_A, LB_DQB0303_A and LB_DQB0303_B we observed multiple uniquely shared residues that were not in a 3.5 Å radius. Therefore, we identified multiple potential functional epitopes that could have triggered the antibody response. It is evident that SAB reactivity analysis of the mAbs with HLA-EMMA resulted in a more extensive analysis of the possible functional epitopes than HLAMatchmaker. Furthermore, some of the eplets identified by HLAMatchmaker exceed the 3.5 Å radius defining an eplet (11), a problem which was also previously addressed by Kramer et al. (35). Interestingly, an earlier version of the HLA Epitope Registry included eplets 45GE_3_ (45G46E47F 74A75V77R 52L53L55L56P57A), 84QL_3_ (84Q85L86E90T 53L 125A) and 52LP_3_ (52P53L 140T 182N) (20), where the subscripted numbers indicated the possible numbers of epitopes that are shared within the same group of HLA-DQ antigens (20). We speculate that these former eplet definitions were removed from the currently available version of the HLA Epitope Registry website because they exceed the 3.5 Å radius. However, these eplet definitions do resemble the reactivity patterns of our generated mAbs and possible include residues that contribute to the actual functional epitope. Additionally, mismatched AAs between the immunizer and antibody producer that were not uniquely shared between the reactive HLA alleles could still be a part of the functional epitope when they are located within 3.5 Å of the uniquely shared residue.

For mAbs LB_DQB0402_A and LB_DQB0602_B, residue 55R was identified as the uniquely shared residue, which ensured the antibody-verification of eplet 55R with our more advanced methodology than the adsorption and elution study currently used for the verification of the eplet (32). Curiously, eplet 55R was not identified by HLAMatchmaker analysis, while this eplet is present in the HLA Epitope Registry. This issue of inaccuracy in eplet software has been previously addressed by Tassone et al. (51). As observed in the SAB data, 55R was also the uniquely shared residue for mAb LB_DQB0601_B, but this mAb demonstrated increased binding strength for eplet 55R with residue 53Q as additional configuration. Since binding and cytotoxicity still occurred in the absence of 53Q, it seems that 53Q is not a crucial AA configuration (18), a phenomenon that was previously described for a number of HLA-DR eplets (35). However, based on the location of the two residues, we cannot rule out that the actual functional epitope that induced the antibody response of LB_DQB0601_B mAb was the combination of residue 55R and 53Q.

Overall, in-depth analysis of luminex SAB data of HLA-specific mAbs can result in the identification of functional epitopes on HLA molecules. However, in some cases further studies of the interaction between antibody and HLA molecule is required to establish the functional epitope that determines the antibody specificity. In future studies, crystallography or cryogenic electron microscopy (cryo-EM) of the binding interface between mAbs and their target HLA will allow for precise localization of the involved residues that interact with the CDR-H3 of the mAb and will decipher the residues that comprise the functional epitope (52). Additionally, site-directed mutagenesis of HLA molecule targets can be used to deduce crucial AAs involved in antibody binding (53) similar as has been done in the past for HLA epitope mapping (54). However, the influence of AA substitution on surface potential has to be considered (55). Finally, testing mAbs LB_DQB0201_A, LB_DQB0303_A and LB_DQB0303_B against a larger panel of cells bearing less common HLA alleles, which are not included in the current SAB assays, could eliminate specific residues as the possible functional epitope, since there are several less common but ‘well-documented’ HLA-DQB1 alleles (56, 57) that contain only some, but not all residues of a reactivity pattern.

Eplet-based HLA matching could be an important step in preventing dnDSA formation and therefore improve graft outcomes after transplantation. Although it was not possible to determine one single functional epitope for several of the HLA-DQ specific mAbs, the reactivity patterns are still relevant and useful for matching strategies in clinical transplantation, because the uniquely shared AAs on the reactive HLA alleles are always present together on the alleles in the currently used SAB assays and other commonly observed HLA-DQB1 alleles (56, 57). The correct definition of eplets, which can be complex as becomes apparent from this investigation and previously performed analyses (35, 58), is crucial for studies that aim to define the immunogenicity of individual eplets, as such investigations will be biased if eplets that actually belong to the same reactivity pattern are considered as multiple separate eplets (59). Consideration of reactivity patterns that consist of multiple eplets or AAs is also of importance when the total eplet or AA mismatch load is used to determine immunological risk, since this method could result in an overestimation of the molecular mismatch when multiple eplet or AAs that belong to a single antibody induction are added up. Furthermore, we acknowledge that simply using the molecular mismatch load as a parameter for immunological risk does not provide a full picture, since dnDSA formation can already be induced by a single eplet or AA mismatch (17). Additionally, alternative algorithms considering physiochemical properties of AA mismatches (12) and the presence of T cell epitopes for indirect presentation (60) may also be of value in the assessment of the risk to develop humoral alloimmunity in the clinical setting.

Although experimental verification of theoretical HLA eplets using HLA-specific mAbs is a laborious endeavor, we consider this as the preferred method of verification of theoretical eplets over other methods such as serum analysis of immunized patients, due to the polyclonal nature of the circulating antibodies. Nevertheless, alternative approaches using clinical datasets can be complementary to the experimental verification of HLA eplets. In this respect, one of the aims of the 18^th^ International HLA and Immunogenetics workshop (https://www.ihiw18.org/) is the definition of immunogenic epitopes by analyzing a large dataset of single antigen bead data from non-sensitized first kidney transplant recipients. Since second-field HLA typing is available for these patients, antibody reactivity can be mapped to known eplets, prompting further experimental verification of these eplets.

To the best of our knowledge, this is the first study in which recombinant human HLA-DQ-specific mAbs were generated through isolation of memory B cells from immunized individuals using biotinylated soluble HLA-DQ monomers. This unique set of mAbs is not only an excellent tool for in-dept analysis of HLA-DQ eplets, but also provides the opportunity to study the pathogenicity of HLA-DQ antibodies, which are the most prevalent alloantibodies in renal transplantation (4–9).

## Data Availability Statement

The raw data supporting the conclusions of this article will be made available by the authors, without undue reservation.

## Ethics Statement

The studies involving human participants were reviewed and approved by the medical ethics committee of Leiden University Medical Center (Leiden, the Netherlands). The patients/participants provided their written informed consent to participate in this study.

## Author Contributions

CK, SH, and FC conceived and designed the study. CK and SB acquired, analyzed, and interpreted the data. The experiments were performed by CK, SB, MF-D, MV, MU-M, and KB. RB provided essential reagents. DR provided HLA typing data. SB, CK, SH, and FC wrote the manuscript. DR, RB, and JF critically reviewed the manuscript. All authors contributed to the article and approved the final version of the manuscript.

## Conflict of Interest

RB is employed by Pure Protein LLC.

The remaining authors declare that the research was conducted in the absence of any commercial or financial relationships that could be construed as a potential conflict of interest.

## Publisher’s Note

All claims expressed in this article are solely those of the authors and do not necessarily represent those of their affiliated organizations, or those of the publisher, the editors and the reviewers. Any product that may be evaluated in this article, or claim that may be made by its manufacturer, is not guaranteed or endorsed by the publisher.

## Abbreviations

Å, angstrom; AA, amino acid; BCM, background corrected mean fluorescence intensity; CDC, complement-dependent cytotoxicity; DSA, donor-specific antibodies; EBV-LCL, Epstein-Barr virus-transformed lymphoblastoid cell line; FACS-XM, flow cytometric cross match; mAb, monoclonal antibody; MFI, mean fluorescence intensity; SAB, single antigen beads.
