# Supplementary material for: HLA-DQ-Specific Recombinant Human Monoclonal Antibodies Allow for In-Depth Analysis of HLA-DQ Epitopes
Source: Front Immunol. 2022 Jan 7;12:761893. doi: 10.3389/fimmu.2021.761893 (PMC8782272; doi:10.3389/fimmu.2021.761893)
Supplement: Supplementary file 1 [file DataSheet_1.pdf]

Supplementary Table 1 - single antigen bead panel used for analysis

DRB1*01:01	DQA1*02:01/DQB1*02:02
DRB1*01:02	DQA1*02:01/DQB1*03:02
DRB1*01:03	DQA1*02:01/DQB1*04:01
DRB1*03:01	DQA1*02:01/DQB1*06:01
DRB1*03:02	DQA1*03:01/DQB1*03:01
DRB1*03:03	DQA1*03:01/DQB1*03:02
DRB1*04:01	DQA1*03:01/DQB1*04:02
DRB1*04:02	DQA1*03:02/DQB1*02:02
DRB1*04:03	DQA1*03:02/DQB1*03:01
DRB1*04:04	DQA1*03:02/DQB1*03:02
DRB1*04:05	DQA1*03:02/DQB1*03:03
DRB1*07:01	DQA1*04:01/DQB1*03:03
DRB1*08:01	DQA1*04:01/DQB1*04:01
DRB1*08:02	DQA1*04:01/DQB1*04:02
DRB1*09:01	DQA1*05:01/DQB1*02:01
DRB1*10:01	DQA1*05:01/DQB1*02:02
DRB1*11:01	DQA1*05:01/DQB1*03:01
DRB1*11:03	DQA1*05:01/DQB1*04:01
DRB1*11:04	DQA1*06:01/DQB1*03:01
DRB1*12:01	DQA1*06:01/DQB1*03:03
DRB1*12:02	DQA1*06:01/DQB1*04:02
DRB1*13:01	DPA1*01:03/DPB1*01:01
DRB1*13:03	DPA1*01:03/DPB1*02:01
DRB1*13:05	DPA1*01:03/DPB1*03:01
DRB1*14:01	DPA1*01:03/DPB1*04:01
DRB1*14:03	DPA1*01:03/DPB1*04:02
DRB1*14:04	DPA1*01:03/DPB1*06:01
DRB1*15:01	DPA1*01:03/DPB1*18:01
DRB1*15:02	DPA1*02:01/DPB1*01:01
DRB1*15:03	DPA1*02:01/DPB1*04:01
DRB1*16:01	DPA1*02:01/DPB1*05:01
DRB1*16:02	DPA1*02:01/DPB1*09:01
DRB3*01:01	DPA1*02:01/DPB1*11:01
DRB3*02:02	DPA1*02:01/DPB1*13:01
DRB3*03:01	DPA1*02:01/DPB1*14:01
DRB4*01:01	DPA1*02:01/DPB1*15:01
DRB5*01:01	DPA1*02:01/DPB1*17:01
DRB5*02:02	DPA1*02:01/DPB1*19:01
DQA1*01:01/DQB1*05:01	DPA1*02:02/DPB1*01:01
DQA1*01:02/DQB1*05:01	DPA1*02:02/DPB1*04:01
DQA1*01:02/DQB1*05:02	DPA1*02:02/DPB1*05:01
DQA1*01:02/DQB1*06:02	DPA1*02:02/DPB1*28:01
DQA1*01:02/DQB1*06:04	DPA1*03:01/DPB1*01:01
DQA1*01:03/DQB1*06:01	DPA1*03:01/DPB1*04:01
DQA1*01:03/DQB1*06:03	DPA1*03:01/DPB1*04:02
DQA1*01:04/DQB1*05:03	DPA1*03:01/DPB1*05:01
DQA1*01:04/DQB1*06:01	DPA1*04:01/DPB1*04:01
DQA1*02:01/DQB1*02:01	DPA1*04:01/DPB1*13:01

Supplementary Figure 1

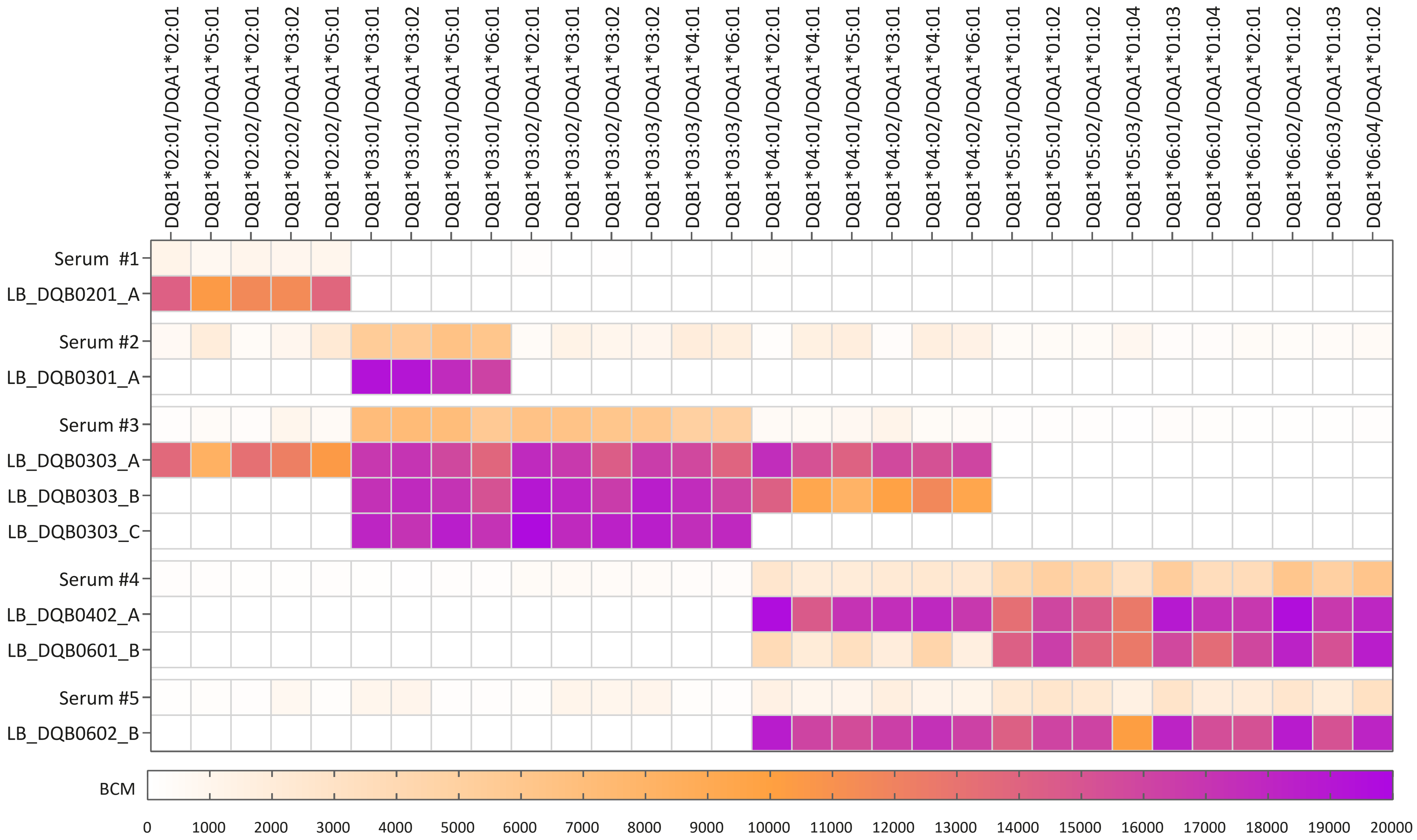

Supplementary Figure 1: HLA-DQ specificities in serum and of recombinant human HLA-DQ monoclonal antibodies. HLA specificity in the serum of the mmunized subjects and HLA specificity of the genereated mAbs as tested in single antigen beads assay. Only HLA-DQ beads are shown as all other loci were negative for the generated mAbs. Recombinant monoclonal antibody concentration tested was 20 µg/ml. BCM: background corrected mean fluorescence intensity.

Supplementary Figure 2

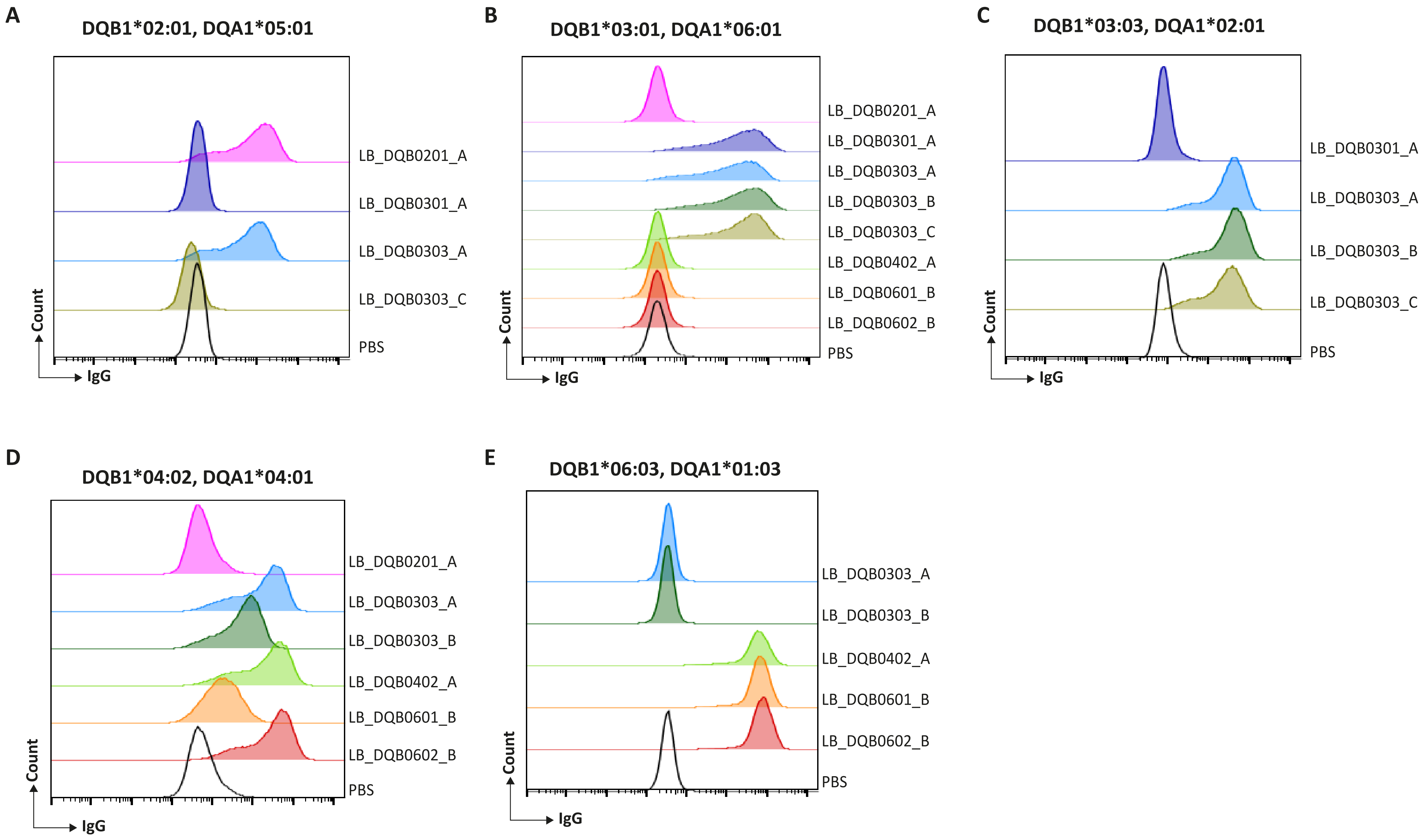

Supplementary Figure 2: Recombinant human HLA-DQ monoclonal antibodies bind to reactive HLA expressed on cells. Flow cytometry crossmatches on Epstein-Barr virus-transformed lymphoblastoid cell lines were performed with each mAb in a concentration of 20 µg/ml. mAb LB_DQB0303_C was tested in a separate experiment under the same conditions.

Supplementary Figure 3

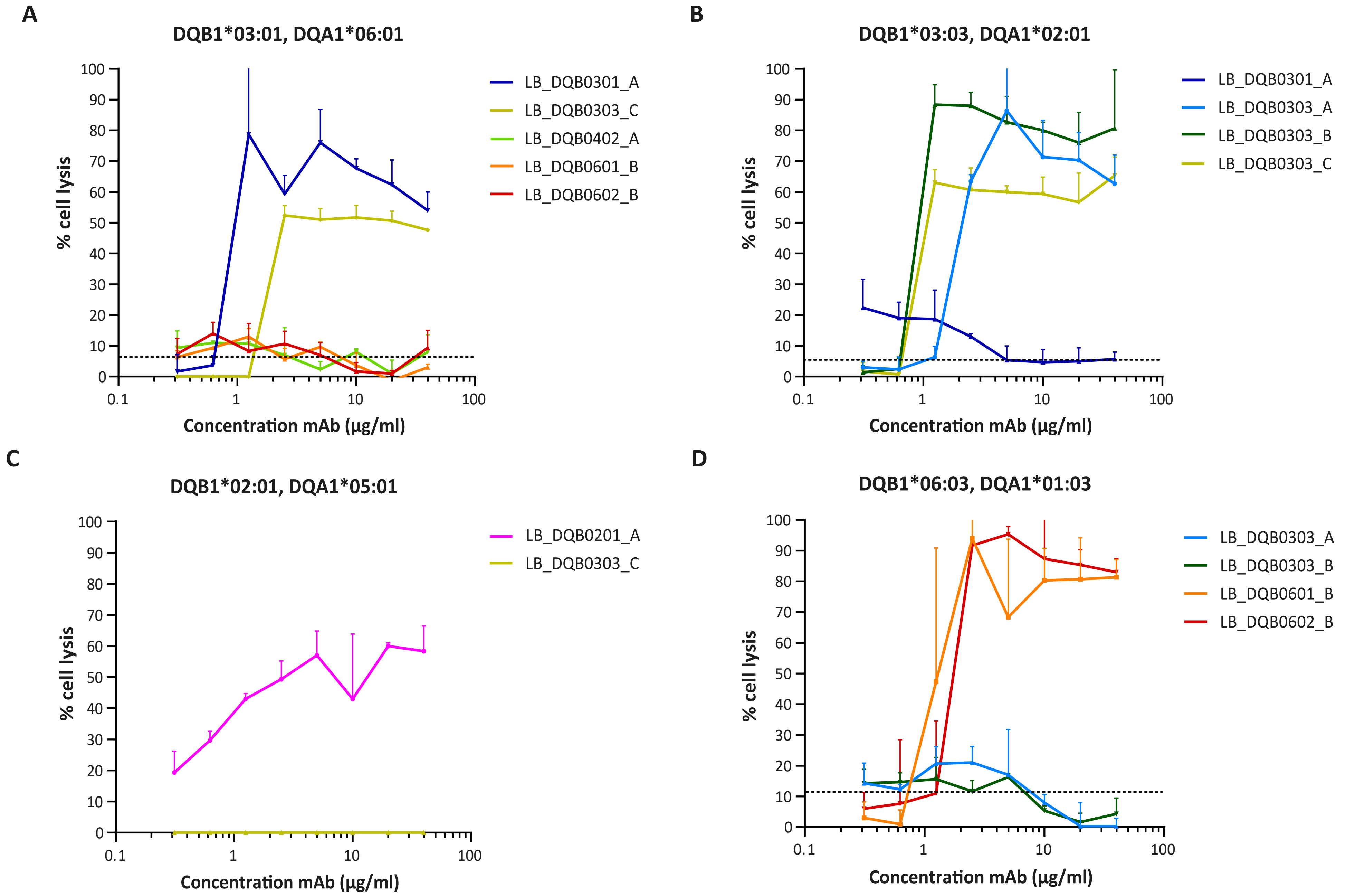

Supplementary Figure 3: Cytotoxicity reactivity of recombinant human HLA-DQ monoclonal antibodies. CDC assays were performed with several HLA-typed Epstein-Barr virus-transformed lymphoblastoid cell lines. mAbs were added in various concentrations: 0.3125, 0.625, 1.25, 2.5, 5, 10, 20 and 40 μg/ml. Data points with error bars represent the mean + standard deviation of triplicate wells. Lysis is relative to the positive control (pan-HLA class II antibody). mAb LB_DQB0303_C was tested in a seperate experiment under the same conditions.

Supplementary Figure 4

LB_DQB0602_B

HLA Allele	BCM	FC-XM	CDC	53	55
**DQB1*06:02/DQA1*01:02**	**18633**			Q	**R**
**DQB1*04:01/DQA1*02:01**	**18575**			L	**R**
**DQB1*06:01/DQA1*01:03**	**18155**			Q	**R**
**DQB1*06:04/DQA1*01:02**	**18051**			Q	**R**
**DQB1*04:02/DQA1*04:01**	**17249**	**Positive**		L	**R**
**DQB1*04:02/DQA1*03:01**	**16290**			L	**R**
**DQB1*04:02/DQA1*06:01**	**16191**			L	**R**
**DQB1*05:02/DQA1*01:02**	**16035**			Q	**R**
**DQB1*04:01/DQA1*04:01**	**15998**			L	**R**
**DQB1*05:01/DQA1*01:02**	**15951**			Q	**R**
**DQB1*04:01/DQA1*05:01**	**15440**			L	**R**
**DQB1*06:01/DQA1*01:04**	**15352**			Q	**R**
**DQB1*06:01/DQA1*02:01**	**15125**			Q	**R**
**DQB1*06:03/DQA1*01:03**	**15037**	**Positive**	**Positive**	Q	**R**
**DQB1*05:01/DQA1*01:01**	**14148**			Q	**R**
**DQB1*05:03/DQA1*01:04**	**10122**			Q	**R**
DQB1*02:01/DQA1*02:01	56			L	L
DQB1*02:01/DQA1*05:01	-85			L	L
DQB1*03:01/DQA1*06:01	-88	Negative	Negative	L	P
DQB1*03:01/DQA1*05:01	-123			L	P
DQB1*03:01/DQA1*03:01	-136			L	P
DQB1*03:03/DQA1*04:01	-174			L	P
Other DQ beads	≤0			L	L/P

Supplementary Figure 4: Reactivity analysis of monoclonal antibody LB_DQB0602_B. Comparison of the amino acid positions of interest of the HLA-DQB1 alleles on the reactive beads in the single antigen bead assay and a selection of the nonreactive HLA-DQB1 alleles. BCM, background corrected mean fluorescence intensity; FC-XM, flowcytometric crossmatch; CDC, complement dependent cytotoxicity
